# *Leishmania donovani* and Cutaneous Leishmaniasis, Sri Lanka

**DOI:** 10.3201/eid1303.060242

**Published:** 2007-03

**Authors:** H. V. Yamuna D. Siriwardana, Harry A. Noyes, Nicholas J. Beeching, Michael L. Chance, Nadira D. Karunaweera, Paul A. Bates

**Affiliations:** * Liverpool School of Tropical Medicine, Liverpool, United Kingdom; † University of Colombo, Colombo, Sri Lanka; ‡ University of Liverpool, Liverpool, United Kingdom

**Keywords:** Parasitic diseases, protozoan infections, leishmaniasis, cutaneous, leishmaniasis, visceral, zoonoses, Sri Lanka, dispatch

## Abstract

To investigate the relationship of cutaneous leishmaniasis isolates from Sri Lanka to known species, we performed DNA sequencing and microsatellite analyses. We identified *Leishmania donovani* as the agent of Sri Lanka cutaneous leishmaniasis and showed that these parasites are closely related to those causing visceral leishmaniasis in the Indian subcontinent.

Infection with *Leishmania* protozoa can result in cutaneous, mucocutaneous, or visceral leishmaniasis (VL), depending on the parasite, host, and environmental factors (*1*). Globally, the disease results in ≈2 million new cases and 2.4 million disability-adjusted life years each year (*2*). The leishmaniases have received renewed interest because of an upsurge of cases in traditionally leishmaniasis-endemic areas and the emergence of new foci of disease (*3*,*4*). One of the most dramatic examples is a new focus of cutaneous leishmaniasis (CL) in Sri Lanka (*5*), from which >400 cases have been reported since 2001.

Previously, multilocus enzyme electrophoresis (MLEE) characterization of a small number of isolates led to the surprising conclusion that CL in Sri Lanka was caused by *Leishmania donovani* (*5*). However, *L. donovani* typically causes VL, a potentially fatal disease and ongoing public health problem in neighboring India, Bangladesh, and Nepal, as well as in East Africa (*1*,*2*). No cases of VL have been reported in Sri Lanka. Occasional cases of CL due to *L. donovani* have been described in other VL-endemic regions (*6*–*9*). Karunaweera et al. (*5*) examined a limited number of isolates and used a single technique, MLEE. Although this technique is usually reliable for characterizing isolates, important exceptions were found in a recent study on *L. donovani* in East Africa (*10*). Therefore, we further investigated Sri Lanka CL by examining more isolates and using 2 molecular techniques.

## The Study

Suspected clinical diagnoses of CL were confirmed by demonstrating the presence of *Leishmania* amastigotes in skin lesions, promastigotes in cultures, or both (*5*). Ethical approval was obtained from the Ethics Review Committee, Faculty of Medicine, University of Colombo. PCR, performed as described (*11*), confirmed 15 primary isolates as members of the genus *Leishmania.* Eight of the Sri Lanka isolates originated from Welioya (northeast), 1 from Jaffna (north), and 2 from Galle (south).

**Figure 1:**
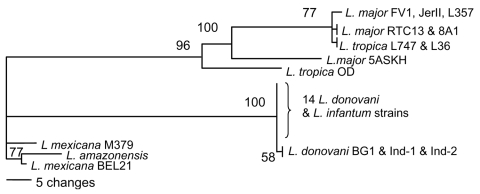
Classification of *Leishmania* species according to the partial DNA sequence of the 6-phosphogluconate dehydrogenase gene constructed with PHYLIP (http://evolution.genetics.washington.edu/phylip.html) using parsimony. Numbers at branch points are bootstrap values compiled by using 100 replicates. Isolates examined and the accession numbers of their 6PGDH sequences in the GenBank/EMBL/DDBJ database are as follows: 11 Sri Lanka isolates, L59, L60, L75, L78, L80, L284, L304, L355, L330, L301, L348 (AJ888888-AJ888898); 2 India isolates, Ind-1, Ind-2 from splenic aspirates of visceral leishmaniasis patients in Muzafapur, Bihar (MHOM/IN/2004/Ind-1 and MHOM/IN/2004/Ind-2, AJ888900, AJ888901); 3 previously identified *L. donovani* isolates BG1 (MHOM/BD/1997/BG1, AJ888899), LEM719 (IMAR/KE/1962/LRC-L57; LEM719, AJ888902), LV9 (MHOM/ET/1967/HU3;LV9, AY168567); and *L. infantum* JPC (MCAN/ES/1998/LEM935;JPC;M5, GeneDB LinJ35.2940). Also analyzed were sequences from the following isolates: *L. tropica* (AY045763, AY168568), *L. major* (FV1, AF242436; 8A1, AF242436; RTC13, AY706106; JerII, AY706105; 5ASKH, AY706107), *L. mexicana* (M379, AY217723; BEL21, AY386372), and *L. amazonensis* (PH8, AY168562).

DNA sequencing of a single-copy gene was used to identify the *Leishmania* species (*10*). The 6-phosphogluconate dehydrogenase (6PGDH) gene was chosen because it shows a high degree of sequence polymorphism among *Leishmania* species (*12*), is well represented in sequence databases, and is known to differentiate the main zymodeme from *L. donovani* in India (MON-2) from that elsewhere (*13*). Primers for conserved regions of 6PGDH were designed by using full-length gene sequences of the *L. major* FV1 (MHOM/IL/1980/Friedlin) and *L. mexicana* BEL21 (MHOM/BZ/1982/BEL21) reference strains. Primers 6PGDH-F (AAT CGA GCA GCT CAA GGA AG) and 6PGDH-R (GAG CTT GGC GAG AAT CTG AC) were designed to generate a 997-bp amplicon incorporating the 822-nt partial 6PGDH sequence that is represented for multiple *Leishmania* species in GenBank. The partial sequences of 6PGDH genes were obtained from 11 Sri Lanka isolates from patients with CL, 2 India isolates from patients with VL, and 2 additional known *L. donovani* strains. These 15 new sequences and 10 publicly available sequences for species belonging to the genus *Leishmania* were used to construct a classification (Figure 1). Of 17 *L. donovani* and *L. infantum* sequences, 14 were >99% identical and could not be separated; the remaining 3 stocks were from India and Bangladesh (Ind-1, Ind-2, and BG1) and clustered together with 58% bootstrap support. Thus, *L. donovani* from Sri Lanka formed a strongly supported group with *L. donovani* and *L. infantum* from Europe and Africa. This group was quite distinct from the group that includes *L. major* and *L. tropica*, which are the parasite species most closely related to *L. donovani* and *L. infantum* and which both cause CL in Africa and Asia. This analysis provided convincing evidence that all 11 Sri Lanka isolates examined were *L. donovani* or *L. infantum*.

Strains of *L. donovani* from Sri Lanka were typed as zymodeme MON-37 by MLEE (*5*). This differs from the predominant India zymodeme (MON-2) in the mobility of 1 isoenzyme, 6PGDH. Therefore, the sequences were further analyzed to investigate the sequence variation underlying the isoenzyme identification. Translation of the 822-nt sequences showed 1 amino acid change that was consistent with the results of MLEE. A single nucleotide difference at position 976 was responsible for the occurrence of an uncharged asparagine (codon AAC) in MON-2 or a negatively charged aspartic acid (codon GAC) in MON-1, MON-18, and MON-37 sequences. This single change would explain the lower mobility of the MON-2 6PGDH isoenzyme, similar to the situation previously reported for glutamate oxaloacetate transaminase isoenzymes in East Africa *L. donovani* strains (*10*).

**Figure 2:**
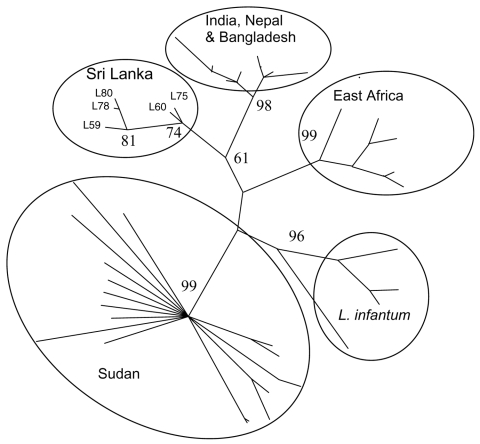
Classification of *Leishmania donovani* and *L. infantum* isolates constructed by using microsatellite data with parsimony in PAUP (Sinauer Associates Inc., Sunderland, MA, USA). Numbers at branch points are bootstrap values compiled by using 100 replicates. Isolates formed geographically based groups (circled). Sri Lanka isolates L59, L60, L75, L78, and L80 are indicated. The tips of other branches are from a dataset of other previously analyzed isolates, including all those identified as *L. donovani* or *L. infantum* and isolates from the Indian subcontinent (*10*).

To more closely analyze the relationships of the *L. donovani* and *L. infantum* strains, we performed microsatellite analysis (*10*). These data were combined with a dataset comprising 40 previously examined *L. donovani* and *L. infantum* isolates (Figure 2). The Sri Lanka isolates clustered together and close to a group containing *L. donovani* isolates from India, Bangladesh, and Nepal. *L. infantum* isolates formed a distinct cluster, as did the *L. donovani* isolates from Sudan and Kenya. This analysis reconfirms recent observations (*10*,*14*) that *L. donovani* isolates tend to cluster on a geographic basis, which suggests that strains of this parasite are geographically distinct. Also, although the Sri Lanka isolates form 1 or possibly 2 distinct groups, they are most closely related to *L. donovani,* which causes VL in India, and distant from *L. infantum* parasites.

## Conclusions

The results of this study led us to conclude that in Sri Lanka, CL is caused by *L. donovani,* which affects the epidemiology and clinical management of leishmaniasis. CL in Sri Lanka can no longer be regarded as a minor problem; an explosion of cases in the past 5 years, undoubtedly an underrepresentation of the true incidence of disease, has not included a single case of VL. However, the possibility that VL will emerge should be considered because subclinical infection is frequent in VL-endemic areas (*15*). The clinical management of CL is often self-cure, which may be preferable to active treatment because self-cure may promote natural immunity to reinfection. Alternatively, antileishmanial drugs may be administered topically or by intralesional injection (*2*). However, *L. donovani* is recognized as one of the great scourges of mankind (*3**,**4*), and if visceral disease does emerge as a problem, more aggressive treatment of CL in Sri Lanka should be considered, e.g., parenteral administration of antimonial compounds, amphotericin, or oral miltefosine. Unfortunately, no drugs are currently registered for the treatment of leishmaniasis in Sri Lanka, and cryotherapy is the only available option in most healthcare centers. Better availability of drugs to treat CL in Sri Lanka is needed, but their introduction must be carefully monitored and critically evaluated.

Our study also raises questions about how infection with apparently identical or very similar parasites can result in radically different types of disease. We speculate that the answers likely lie with the nature of the parasites, the genetics of the human population, or the contribution of sandfly vectors. The data presented here demonstrate the overall close genetic similarity among all *L. donovani* isolates examined. However, some critical genetic difference in Sri Lanka parasites may exist and render them less virulent than *L. donovani* from elsewhere. Clearly much work remains to be done, including PCR or serologic investigation of possible subclinical VL, to understand the factors behind the emergence of Sri Lanka CL due to *L. donovani.* Future studies must be a priority as the number of cases continues to increase.
